# High Medial Longitudinal Arch of the Foot and Latent Trigger Points in Lower Limb Muscles

**DOI:** 10.3390/jcm13144049

**Published:** 2024-07-11

**Authors:** Juan Carlos Zuil-Escobar, José Antonio Martín-Urrialde, Antonia Gómez-Conesa, Carmen Belén Martínez-Cepa

**Affiliations:** 1 Departamento de Fisioterapia, Facultad de Medicina, Universidad San Pablo-CEU, CEU Universities, Urbanización Montepríncipe, 28660 Boadilla del Monte, Spain; jamurria@ceu.es; 2 Research Group Research Methods and Evaluation in Social Sciences, Mare Nostrum Campus of International Excellence, University of Murcia, 30100 Murcia, Spain; agomez@um.es

**Keywords:** medial longitudinal arch, lower extremity, trigger point

## Abstract

**Background:** The objective was to evaluate the prevalence of latent trigger points (LTrPs) in lower limb muscles in participants with a high medial longitudinal arch (MLA) of the foot compared to controls. **Methods**: Participants with a navicular drop test of 4–9 mm were included in the control group; the high MLA group included navicular drop test values of ≤4 mm. The presence of LTrPs was assessed by palpation techniques. The muscles evaluated were medial gastrocnemius (LTrP1), lateral gastrocnemius (LTrP2), soleus (LTrP1), peroneus longus, peroneus brevis, tibialis anterior, extensor digitorum longus, flexor digitorum longus, rectus femoris, vastus medialis (LTrP1 and LTrP2), and the vastus lateralis of the quadriceps (LTrP1 and LTrP2). **Results**: Thirty-seven participants with high MLA and thirty-seven controls were included in the study. Twenty-nine (78.4%) participants in the high MLA group had at least 1 LTrP, compared to twenty-three (62.2%) in the control group. No statistical difference (*p* < 0.05) was found in the total number of LTrPs between groups (4.46 ± 3.78 vs. 3.24 ± 3.85). There were more participants (*p* < 0.05) with LTrPs in the tibialis anterior, extensor digitorum longus, and vastus lateralis (LTrP1 and LTrP2) in the high MLA group than in the control group. **Conclusion**: Although no differences were found in the number of total LTrPs between groups, the prevalence was statistically significantly higher in the tibialis anterior, extensor digitorum longus, and vastus lateralis of the participants with high MLA of the foot.

## 1. Introduction

The medial longitudinal arch (MLA) of the foot has important functions in both standing and walking and shows variations in its height [1]. A high MLA is less flexible than a standard arch [2,3] and is associated with varus [2] and supination of the rearfoot [4]. Individuals with a high MLA of the foot have reduced foot mobility [1], greater leg stiffness [4], reduced shock absorption [3,5] and increased peak plantar pressure [6]. These individuals with cavus foot have lower plantar pressure at the MLA and increased plantar pressure at the forefoot and heel compared to those with normal or low MLA of the foot [1,6,7,8,9]. High MLA of the foot also affects the kinematics of the lower limb during walking and running; it is associated with lower knee flexion excursion [4], smaller peak knee abduction movements [10], shorter contact times, and smaller centre of pressure excursion [4]. 

Variations in the alignment are risk factors for lower limb injuries [11] and changes in the height of the foot’s MLA are associated with a greater prevalence of injuries [12,13], including plantar fasciitis [2,14], knee flexor injuries [2], triceps surae injuries [2], non-contact anterior cruciate ligament injuries [15], lateral ankle sprains [16], foot pain [6] and bony injuries [5]. Feet with a high MLA are more likely to suffer stress fractures, [12] particularly in the tibia, femur [17] and fifth metatarsal [5]. Although both increased and decreased arches are associated with lower limb injury; the strength of this relationship is weak [18]. In addition, a high MLA of the foot affects muscle function. The strength of the ankle dorsiflexion muscles is lower in participants with cavus foot compared to controls [19] and high-arch runners show significantly earlier electromyographic onset of vastus lateralis (VL) compared to low-arch runners [4]. However, the height of the MLA does not affect both the vertical jump and the standing long jump [20]. An increased MLA, therefore, makes the foot a stiffer structure, which, as mentioned above, may be associated with the development of injury and may also affect gait and running.

There are different methods to evaluate the height of the MLA of the foot, including radiographic parameters, footprint angles, or indexes and clinical tests. Although the radiography evaluation is the gold standard for assessing the medial longitudinal arch, its negative side effects, such as ionizing radiation, make its use impractical and unjustified [18]. Clinical tests include the navicular drop test (NDT) [21], described by Brody [22], which assesses the excursion of the navicular tuberosity in the sagittal plane in two positions: the subtalar neutral position unloaded and the relaxed position under load. The NDT is an inexpensive method of assessing the height of the MLA and has shown high reliability in both healthy individuals [23,24] and those with lower limb injuries, such as patellofemoral syndrome [23] or rheumatoid arthritis [25]. In addition, the NDT has shown significant correlations with footprint parameters [26,27,28]. Values between 5 and 9 mm are usually considered normal, with higher values associated with low MLA and lower values associated with high MLA [29]. NDT has been used to assess the height of the MLA, including in both high and low arches [30], showing significant effect sizes in identifying both high and low MLA [18].

A myofascial trigger point is a hyperirritable focus in a taut band of muscle that is painful when the muscle is compressed, stretched, or overstretched [31]. They can be classified as active trigger points (ATrPs) or latent trigger points (LTrPs) depending on the presence or absence of spontaneous pain, respectively [32]. LTrPs cause changes in ultrasound imaging [33], biochemical changes [34], and spontaneous electrical activity [35], in addition to affecting reciprocal inhibition [36] and causing muscle spasm [37]. They also decrease strength [31,38], reduce the range of motion (ROM) [35], and affect muscle activation patterns [39]. In addition, LTrPs can become ATrPs [31]. Therefore, both assessment and treatment of LTrPs may be necessary in clinical practice. They can be present in both patients with myofascial pain syndrome [40,41] and pain-free subjects [42,43]. For example, 77.7% of pain-free subjects have at least one LTrP in the lower limb muscles [44], with a prevalence ranging from 13% to 37.4% [44,45]. 

There are many causes associated with the development of LTrPs, and changes in posture and joint alignment have been suggested as one of these factors [31,46]. Therefore, previous studies have associated the presence of trigger points with head extension and reduction in cervical lordosis in migraineurs patients [47], as well as forward head posture [48]. With regard to changes in lower limb alignment and the presence of LTrPs, it has previously been shown that people with a reduced internal longitudinal arch have a greater number of LTrPs than people with a normal-height MLA [49]. However, the relationship between a higher MLA and LTrPs has not been investigated, and no previous studies have compared the prevalence of LTrPs in subjects with a high MLA to controls. Our hypothesis is that the presence of a high MLA is associated with a higher prevalence of latent trigger points.

The primary objective of this study was to assess the prevalence of LTrPs in several lower limb muscles in participants with a high MLA of the foot compared to controls. Secondly, the intra-rater reliability of the navicular drop test (NDT) and the diagnosis of LTrPs were calculated. 

## 2. Materials and Methods

### 2.1. Study Design 

A cross sectional study was carried out. 

### 2.2. Sample Size 

The first 40 participants (20 with a high MLA of the foot and 20 controls) were used to calculate the sample size of the study and the intra-rater reliability of the procedures. The prevalence of the LTrPs located in the ankle dorsiflexors and VL was used to calculate the sample size. The Ene programme (version 3.0) was used with a precision level of 5% and 80% power. The sample size obtained was 37 participants per group. 

### 2.3. Intra-Rater Reliability

With regard to the intra-rater reliability of the NDT and LTrPs diagnosis, a test-rest was carried out, with a period of 48 h between assessments [50,51]; the rater was a physiotherapist with 20 years’ experience. 

### 2.4. Participants and Settings

Pain-free volunteers who responded to an e-mail campaign were included in the study. A convenience sampling method was used. This sample has some limitations: it may not be representative of the population as a whole, and the results cannot be extrapolated to the general population, which limits its external validity [52]. Participants were recruited from the staff and students at the University of San Pablo-CEU. The project observed the principles outlined in the Declaration of Helsinki of 1975, revised in 1983, and it was evaluated by the Research Ethics Committee of the CEU-San Pablo University. All participants were informed of the aims and procedures of the study and completed an informed consent form before being included in the research. Inclusion criteria included an NDT ≤ 4 mm in the high MLA of the foot group and an NDT between 5 and 9 mm in the control group [24]. Participants were excluded if they had undergone lower limb surgery, had acute lower limb injuries, had lower limb deformities, had systemic or neurological conditions that could affect pain perception, and/or had a reduction in lower limb ROM compared to normal. The ball kick test was used to determine lower limb dominance [53].

### 2.5. Variables

The variables assessed were:

- Demographics: sex, height, weight, and body mass index.
- Type of MLA: standard (NDT from 5 to 9 mm) or high (NDT ≤ 4 mm) [24].
- Total number of LTrPs in all muscles assessed.
- Prevalence of LTrPs in each of the muscles assessed.

### 2.6. Measurement

**Figure 1:**
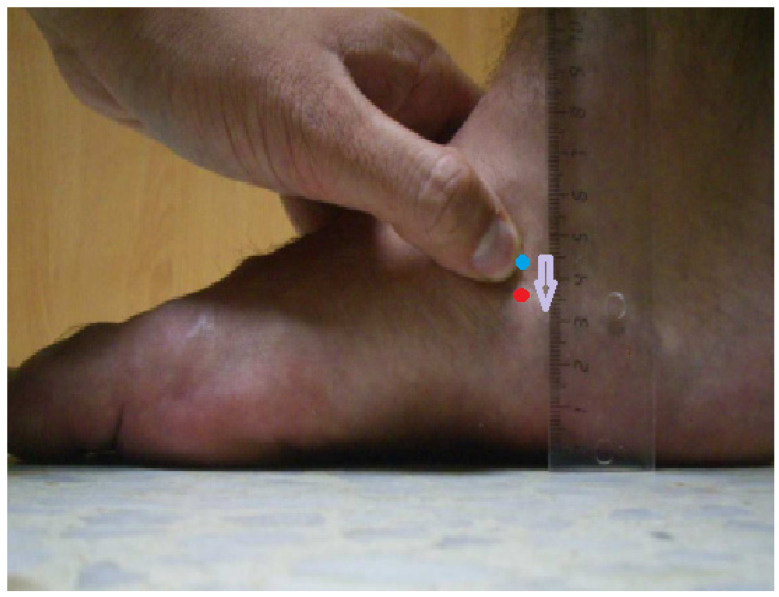
Navicular drop test. The blue dot is the navicular tuberosity with the subtalar joint in the neutral position, while the red dot is the navicular tuberosity with the foot relaxed. The arrow shows the movement of the navicular during the test.

The assessment of the MLA was performed prior to the assessment of LTrPs by a physiotherapist with more than 20 years of experience in using the test. A modification of the Brody [22] procedure was used: with the subject standing barefoot on the floor, the navicular tuberosity was marked. The examiner palpated the medial and lateral aspects of the talar dome, with the thumb over the talar sinus and the index finger over the anteromedial portion. The foot was then slowly inverted and everted until the depressions felt under both fingers were equal. With the subtalar joint in neutral position, the distance between the navicular tuberosity and the ground was measured (in millimeters) using a ruler. The height of the navicular tuberosity was then measured again with the foot in a relaxed position. The NDT was the difference between the two measurements [22]. The procedure was repeated three times, and the average was recorded (Figure 1).

Palpation techniques were used to assess the prevalence of LTrPs [31]. The identification of a LTrP was considered positive if 2 or more [45] of the following criteria [31] were present:

- A palpable taut band in skeletal muscle.
- A hypersensitive tender spot.
- The reproduction of referred pain in response to compression.
- The jump sign.
- A local twitch response provoked by palpation of the taut band.

The following muscles were assessed [54]: gastrocnemius (GM) (LTrP1 and LTrP2), soleus (LTrP1), peroneus longus (PL), peroneus brevis, tibialis anterior (TA), extensor digitorum longus (EDL), flexor digitorum longus, rectus femoris (RF), vastus medialis (VM) (LTrP1 and LTrP2), and VL (LTrP1 and LTrP2). Flat palpation was used on the quadriceps muscles, with the participant in the supine position. For palpation of the VL, the knee was extended. For the palpation of the RF, the lower limb was placed in moderate abduction with the knee extended, while for the VM, the knee was flexed 90°. Flat palpation in the supine position was also used to examine the TA, EDL, and both peroneals. The flexor digitorum longus was assessed by flat palpation with the patient in a side-lie position. Pincer palpation was used to assess the GM, with the participant in the lateral decubitus position. Flat palpation was used for the soleus, with the participant in the lateral decubitus position with the knee flexed. The order in which the LTrPs were assessed was randomized for each subject. The LTrPs were assessed by a physiotherapist with 20 years’ experience in the management of myofascial pain syndrome. Participants were blinded to the results of both the NDT and LTrPs assessments. 

### 2.7. Statistical Methods

Statistical analysis was performed using IBM SPSS 24, with an alpha level of 0.05 for all the tests performed. The Kolmogorov–Smirnov test was used to assess the normal distribution of the quantitative variables; in this case, parametric tests were performed. Descriptive analysis was performed using means and standard deviations for quantitative variables, and frequencies and percentages for qualitative variables. The intra-rater reliability of the NDT (continuous variable) was assessed using the intraclass correlation coefficient (ICC), and Cohen’s kappa was used to assess the diagnosis of the LTrPs (non-continuous variable). The ICC was determined by using mixed-effect and absolute agreement or consistency 2-factor alpha models. Differences in quantitative demographic variables and the number of LTrPs between the two groups were assessed using the unpaired Student *t* test. The chi-squared test was used to assess the difference in qualitative demographic variables and the prevalence of LTrPs in each muscle between the two groups.

## 3. Results

### 3.1. Prevalence of LTrPs

#### 3.1.1. Participants and Descriptive Data

The high MLA foot group included 21 women (56.8%) and 16 men (43.2%), whereas the control group included 22 women (59.5%) and 15 men (40.5%). Table 1 shows the characteristics of the participants. There were no statistically significant differences (*p* > 0.05) in demographic variables between the groups.

#### 3.1.2. Main Results

In the high MLA group, twenty-nine (78.4%) participants had at least 1 LTrP, compared to 62.2% (twenty-three participants) in the control group. Although the high MLA foot participants had more LTrPs (mean: 4.46 ± 3.8) than the controls (mean: 3.24 ± 3.9), no statistical difference was found in the unpaired Student *t* test (*p* > 0.05). 

**Figure 2:**
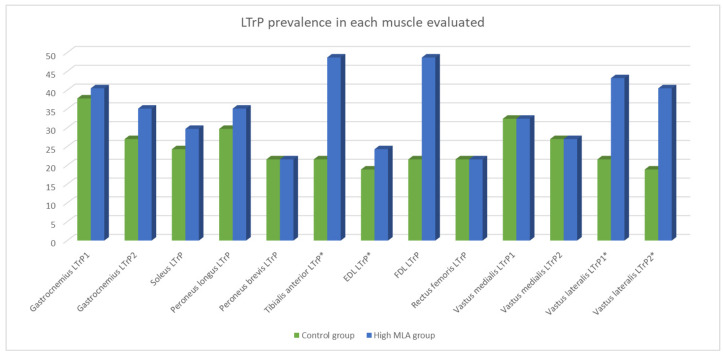
Prevalence (%) of LTrPs in high MLA and control groups. Statistical differences (*p* < 0.05) are found in tibialis anterior, extensor digitorum longus and vastus lateralis. * = *p* < 0.05.

The Chi-squared test showed statistically significant differences (*p* < 0.05) in the prevalence of LTrPs in the TA, EDL, and both VLs (LTrP1 and LTrP2). The high MLA group had a higher number of LTrPs in these muscles than the control group. Table 2 and Figure 2 show the prevalence of each LTrP.

### 3.2. Intra-Rater Reliability

There were 11 women (55.5%) in the high MLA foot group and 10 (50%) in the control group. Both groups showed excellent intra-rater reliability for the NDT. The ICC (2,1) was 0.957 (95% confidence interval, 0.895–0.983) in the control group and 0.959 (95% confidence interval, 0.899–0.983) in the high MLA group. The intra-reliability of the diagnosis of LTrPs was excellent in both the control group (0.828–1) and the high MLA group (0.828–1). Table 3 shows all the values. 

## 4. Discussion

The main objective of this study was to assess the prevalence of LTrPs in different muscles of the lower limbs in subjects with an increased MLA compared to controls. Although no significant differences were found in the total number of LTrPs between the groups, participants with increased MLA had a higher number of LTrPs in the TA, ED, and VL. The secondary objective was to assess the intra-observer reliability of the NDT and the diagnosis of LTrPs, both of which were excellent.

### 4.1. Intra-Rater Reliability

The reliability of clinical tests is important to researchers and clinicians [55], as it is important for the interpretation of the measurements, whereas low reliability may be a source of bias. In this study, the intra-rater reliability for the NDT was excellent in both the control and high MLA groups. Good to excellent reliability, both intra-rater [23,25,50,56,57] and inter-rater [23,25,50,51,56], has been demonstrated previously. In participants with a low foot MLA, the intra-rater reliability for the NDT was also excellent [51]. The intra-rater reliability of the LTrP diagnosis was excellent (0.828–1). The reliability of the LTrP diagnosis in the lower limb muscles has been previously studied and ranged from moderate to excellent [44,49,58,59,60,61]. Palpation techniques are currently the preferred method for the clinical diagnosis of LTrPs, and reliable palpation is required for a diagnosis to be considered valid [61]. Reliability is related to the experience of the rater [58], palpatory skills [61], and the location of the muscles [61].

### 4.2. Prevalence of LTrPs

The main goal of this study was to evaluate and compare the prevalence of LTrPs in the lower limb muscles in both participants with high MLA of the foot and controls. There are no previous studies that have evaluated this. Although participants with high MLA had a higher number of LTrPs (mean: 4.46 ± 3.8) than controls (mean: 3.24 ± 3.9), there was no statistical difference. However, significant differences were found in some of the muscles examined. The high MLA group showed a higher prevalence of LTrPs (*p* < 0.05) in the TA, EDL, and VL (LTrP1 and LTrP2) compared to the control group. Previous researchers have compared the prevalence of LTrPs in controls and participants with other clinical characteristics. Zuil-Escobar et al. [49] compared participants with low MLA of the foot (n = 82) and controls (n = 82), and found no statistical differences in the total number of LTrPs between the two groups. However, the low MLA foot group showed a higher prevalence of LTrPs than the control group (*p* < 0.05) in the TA, FLD, and VM. In the first group, the prevalence of LTrP in these muscles ranged from 38% to 43%, whereas in the control group, it ranged from 18% to 26%. Torres-Chica et al. [62] evaluated the prevalence of both ATrPs and LTrPs in post-meniscectomy pain participants and controls in the GM, RF, VM, and VL. They found no statistical difference in the total number of LTrPs between the groups. The prevalence of LTrPs ranged from 3% to 63.3% in the control group and from 15.2% to 51.5% in the post-meniscectomy pain group. In both groups, the highest prevalence was found in the GM and the lowest in the RF. Bajab et al. [63] compared the prevalence of both ATrP and LTrP in the quadriceps, triceps surae, and the peronei in osteoarthritis participants (n = 14) and controls (n = 14). The osteoarthritis group had a higher prevalence of LTrPs than controls (*p* < 0.05), with LTrPs present in all muscles examined (7.1%-64.3%). In participants with knee osteoarthrosis, Sánchez-Romero et al. [64] found a prevalence of LTrPs ranging from 12% to 50% in different lower limb muscles, including quadriceps and gastrocnemius. Rozenfeld et al. [65] found differences in the prevalence of both ATrPs and LTrPs in the VM, VF, and RF in military personnel with anterior knee pain (n = 65) and controls (n = 24). While only 6.3% of controls had at least 1 ATrP or LTrP, 78.8% of the participants with anterior knee pain had at least 1 ATrP or LTrP. In addition, other studies have shown that LTrPs in the lower limb are common in pain-free subjects [44,45,66]. 

Several factors associated with the presence of myofascial trigger points have been hypothesised. One of them may be mechanical dysfunctions [31,54,67]. A lower MLA of the foot and subtalar pronation could be an activating or perpetuating factor for LTrPs located in the VM, PL, peroneus brevis, flexor digitorum longus, and tibialis posterior [54]. Although previous research has shown a greater prevalence of LTrPs in the flexor digitorum longus, TA, and VM in participants with low MLA compared to controls [49], no previous studies have evaluated the relationship between a high MLA and LTrPs. Biomechanical factors may be related to the higher prevalence of LTrPs in the high MLA foot participants compared to controls. A high MLA is associated with calcaneal inversion and forefoot varus [68] and is stiffer than a low or standard MLA [4,69]. Cavus feet have reduced foot mobility [1] and are associated with lower limb stiffness [4] and a reduction in shock-absorbing capacity [3,5,69]. Previously, a high MLA foot has been shown to correlate with reduced ankle dorsiflexion [67,70] and a shortened Achilles tendon [71]. It has been proposed that a low ankle ROM is associated with myofascial trigger points located in the quadriceps muscles [54]. A decrease in ankle dorsiflexion ROM could stretch the plantar flexors, affecting the LTrPs located in the triceps surae [54]. The TA and EDL could be affected simultaneously, as they are agonists of the triceps surae [54]. However, our study did not assess ankle ROM, so it is not possible to conclude whether there is a relationship between ankle ROM and the prevalence of LTrPs in the muscles assessed. In addition to changes in joint biomechanics, the height of the MLA could influence muscle activity and its characteristics [68]. Foot type explains the variation in the anteroposterior thickness of the TA tendon (7.1%), PL muscle (7.6%), and Achilles’ tendon (16%) [72] and is correlated with toe flexor [73,74] and tibialis posterior strength [74]. These changes could be related to the presence of LTrPs in the affected muscles. 

A high MLA of the foot also affects the gait. Therefore, a greater vertical load has been found in participants with high MLA [4], and the speed of the centre of pressure is also affected [75]. During walking and running, people with high MLA of the foot have a different pattern of movement compared to those with a low or normal MLA [10,76,77,78]. In addition, an earlier electromyography onset of VL has been found in participants with high MLA compared to those with low MLA during running [4]. These changes could lead to fatigue in the affected muscles and could be a cause for the presence of LTrPs. During gait, the rotational stress in the weight-bearing lower limb is related to the supination or pronation of the foot during the stance phase, and the movement of the hip, knee, and subtalar joints during this phase is interdependent [70]. Individuals with high MLA of the foot have altered angles in the transverse and frontal planes of the rearfoot [1,10,77,79] and less ROM in the midfoot in both the sagittal and transverse planes during the initial contact and midstance phases [77]. This may be related to the stiffness of the MLA and an increase in vertical loading. It has been suggested that small angular variations in the ROM of the foot may be critical in the development of lower limb overuse injuries [77]; LTrPs may be involved in these injuries. It should also be considered that the NDT may not reflect the dynamic behaviour of the navicular. The limit value for flat feet in dynamic NDT has been set at 8.5 mm [80], which is close to the 10 mm established for the NDT. No reference values for dynamic NDT have been found for high MLA. It should be noted that the NDT does not overestimate the walking motion of the navicular in the hypomobile feet [81], which corresponds to the lowest static NDT values and therefore to the MLAs catalogued as high in our research. This can be seen in the greater joint congruence and stiffness of the soft structures that appear in this type of foot [81]. However, it should also be remembered that the foot is loaded twice as much in monopodal stance as in bipodal clinical testing [82]. It is therefore necessary to include dynamic gait analysis and other imaging tests that can provide complementary information on the dynamic function of the foot compared to the static function assessed by NDT.

Our results suggest that LTrPs are common in the lower limb muscles of both participants with high MLA of the foot and controls. The high MLA group showed a higher prevalence in the ECD, TA, and VL. This high prevalence may be clinically important and the assessment and management of the height of the MLA of the foot may be important in the management of lower limb myofascial pain syndrome. The LTrPs are present in lower limb disorders such a knee osteoarthritis [63,64,66], post-meniscectomy pain [62], calf pain [83], patellofemoral pain syndrome [84], and anterior knee pain [65]. LTrPs do not cause spontaneous pain, but they can affect multiple muscle functions. For example, they can affect muscle activity [85], reciprocal inhibition [36], and muscle activation patterns, reducing overall movement efficiency [39]. They also increase intramuscular electromyographic activity in synergist muscles [86], accelerate muscle fatigability [87], and decrease strength [31,38]. In the lower limb, they can also reduce ankle ROM [88] and increase muscle spasm [37]. LTrPs can easily convert into ATrPs if the underlying causes are not treated [31]. LTrPs are common in different conditions involving biomechanical changes in the lower limb [49,62,63,64,65,66,84] and a high MLA is associated with several injuries [89]. In addition, the height of the MLA is associated with changes in the lower limb biomechanics [4,10,77,90,91]. Although the mechanisms linking foot position and increased risk of lower limb injury are unclear, changes in lower limb biomechanics are considered to be among them [92]. Changes in joint alignment may be one of the causes of the development of LTrPs in these muscles [31,46]. Therefore, the mechanisms involved in the higher prevalence of LTrPs in different muscles in participants with high MLA of the foot should be investigated. Prospective research is needed to investigate the relationship between LTrPs and high MLA of the foot to determine whether the LTrPs located in these muscles are a consequence of the biomechanical changes associated with a cavus foot. Assessment and, where appropriate, control of high MLA may be useful in the management of LTrPs. Control of MLA height has previously been shown to influence the electromyographic activity of lower extremity muscles such as the tibialis anterior, tibialis posterior [93,94], peroneus longus [95,96], and medial gastrocnemius [97]. It may also be helpful to investigate the effect of techniques to control the height of the MLA, such as orthotics or functional taping, on the presence of LTrPs, but it should be noted that there may be other factors associated with the presence of LTrPs in the lower limb musculature, so an individual assessment of each patient is required.

### 4.3. Limitations

This study has limitations. Firstly, this research includes LTrPs; future studies should include ATrPs as they could be associated with foot and ankle pathologies [98], and their study would be interesting. In addition, we did not include all the important muscles of the lower limb, such as the tibialis posterior, the adductors, or the hamstrings. With regard to the arch, we did not differentiate between rigid and mobile high MLA. Midfoot mobility during walking and running is thought to play a role in lower limb function, and arch mobility influences rearfoot movement [99]. This factor may influence the development of LTrPs in lower limb muscles. The height of the MLA was assessed using the NDT. It should be noted that the gold standard for assessing MLA height is radiographic parameters [21]. The NDT has been shown to be less reliable for inexperienced raters [56]. This may be related to the difficulty in locating the navicular tuberosity [100] and positioning the subtalar joint in a neutral position [101]. However, in our study, the assessor was experienced in the use of this clinical test. We sought to limit the risk of bias inherent in the NDT as a clinical test by assessing MLA height prior to the presence of LTrPs. It should also be considered that the NDT may not reflect the dynamic behaviour of the navicular bone and that this study assessed the MLA in static rather than dynamic conditions. Future research is therefore needed to evaluate the dynamic behaviour of the MLA and its relationship to the presence of LTrPs. The use of dynamic NDT can be compromised by the need to use video systems, which can be unreliable and expensive [80]. Finally, a cohort of pain-free young people was studied, so the results cannot be generalized to other populations. Further studies in populations with different demographic characteristics or foot pathology are needed in order to extrapolate the results of this research.

## 5. Conclusions

No significant differences were found in the total number of LTrPs between the participants with high MLA of the foot and the control group. However, the specific prevalence of LTrPs in the TA, EDL, and VL was statistically significantly higher in the high MLA group. The reliability of both NDT and the diagnosis of the LTrPs was excellent. It is necessary to investigate whether both alignment and kinematics in the high MLA of the foot are related to the presence of LTrPs.

## Figures and Tables

**Table 1:** Demographic variables.

	Control Group (n = 37)	High MLA Group (n = 37)	*p* Value
Age	22.84 ± 2.57	23.24 ± 2.63	0.411
Height	169.78 ± 10.70	168.99 ± 10.32	0.794
Weight	67.34 ± 12.88	64.99 ± 11.05	0.133
BMI	23.12 ± 1.97	22.85 ± 1.67	0.288

Characteristics of the participants. Values are means and standard deviations. BMI = body mass index.

**Table 2:** Prevalence of LTrPs.

	Control Group (n = 37)	Higher MLA Group (n = 37)
Gastrocnemius LTrP1	14 (37.8%)	15 (40.5%)
Gastrocnemius LTrP2	10 (27%)	13 (35.1%)
Soleus LTrP	9 (24.3%)	11 (29.7%)
Peroneus longus LTrP	11 (29.7%)	13 (35.1%)
Peroneus brevis LTrP	8 (21.6%)	8 (21.6%)
Tibialis anterior LTrP *	8 (21.6%)	18 (48.6%)
Extensor digitorum longus LTrP *	8 (21.6%)	18 (48.6%)
Flexorum digitorum longus LTrP	7 (18.9%)	9 (24.3%)
Rectus femoris LTrP	8 (21.6%)	8 (21.6%)
Vastus mediales LTrP1	12 (32.4%)	12 (32.4%)
Vastus mediales LTrP2	10 (27%)	10 (27%)
Vastus lateralis LTrP1 *	8 (21.6%)	16 (43.2%)
Vastus lateralis LTrP2 *	7 (18.9%)	15 (40.5%)

Prevalence of LTrPs (frequencies and percentages) in the muscles evaluated. * = *p* < 0.05. LTrP: latent trigger point.

**Table 3:** Intra-rater reliability.

	Control Group (n = 37)	Higher MLA Group (n = 37)
Gastrocnemius LTrP1	1	0.894
Gastrocnemius LTrP2	1	1
Soleus LTrP	0.875	0.886
Peroneus longus LTrP	1	1
Peroneus brevis LTrP	0.875	0.828
Tibialis anterior LTrP	0.828	1
Extensor digitorum longus LTrP	0.828	1
Flexorum digitorum longus LTrP	0.828	0.875
Rectus femoris LTrP	1	0.857
Vastus mediales LTrP1	0.875	0.886
Vastus mediales LTrP2	0.875	0.875
Vastus lateralis LTrP1	0.828	0.898
Vastus lateralis LTrP2	0.828	1

Kappa’s Cohen for diagnosis criteria of latent trigger points LTrPs = latent trigger point.

## Data Availability

All relevant data are contained within the article. The original contributions presented in the study are included in the article. Further inquiries can be directed to the corresponding author.
